# Limited evidence for common interannual trends in Baltic Sea summer phytoplankton biomass

**DOI:** 10.1371/journal.pone.0231690

**Published:** 2020-04-30

**Authors:** Jennifer R. Griffiths, Sirpa Lehtinen, Sanna Suikkanen, Monika Winder

**Affiliations:** 1 Department of Ecology, Environment, Plant Sciences, Stockholm University, Stockholm, Sweden; 2 Finnish Environment Institute, Marine Research Centre, Helsinki, Finland; Universidade de Aveiro, PORTUGAL

## Abstract

The Baltic Sea summer phytoplankton community plays an important role in biogeochemical cycling and in the transfer of energy through the food web via zooplankton. We aimed to improve the understanding of the degree to which large-scale versus local environmental dynamics regulate phytoplankton dynamics by analyzing time series at the Baltic Sea scale. We used dynamic factor analysis to study if there are common patterns of interannual variation that are shared (“common trends”) among summer phytoplankton total and class-level biomass time series observed across Baltic Sea latitudinal gradients in salinity and temperature. We evaluated alternative hypotheses regarding common trends among summer phytoplankton biomass: Baltic Sea-wide common trends; common trends by geography (latitude and basin); common trends differing among functional groups (phytoplankton classes); or common trends driven by both geography and functional group. Our results indicated little support for a common trend in total summer phytoplankton biomass. At a finer resolution, classes had common trends that were most closely associated with the cryptophyte and cyanobacteria time series with patterns that differed between northern and southern sampling stations. These common trends were also very sensitive to two anomalous years (1990, 2008) of cryptophyte biomass. The Baltic Sea Index, a regional climate index, was correlated with two common class trends that shifted in mean state around the mid-1990s. The limited coherence in phytoplankton biomass variation over time despite known, large-scale, ecosystem shifts suggests that stochastic dynamics at local scales limits the ability to observe common trends at the scale of monitoring data collection.

## Introduction

Characterizing the dynamics of phytoplankton biomass in space and time improves our ability to evaluate how shifts in this basal resource may propagate up the food web or alter the dynamics of biogeochemical cycles. Phytoplankton fuel secondary production in aquatic food webs and account for ~50% of net global primary productivity [1]. Additionally, phytoplankton are central to global biogeochemical cycles, contributing ~40% of the carbon fixed annually [2]. Phytoplankton also play important roles in the cycling of silica [diatoms, 3] and nitrogen [4–6] among other elements.

Phytoplankton are known to exhibit a wide array of regular and irregular biomass patterns spanning a large range of spatial and temporal scales [7, 8]. Even in temperate systems, commonly thought to be dominated by strong seasonal dynamics, phytoplankton communities vary widely in their temporal frequencies [9]. Low-frequency variation in phytoplankton biomass has been observed in response to large-scale ocean-climate oscillations [10, 11] in addition to long-term directional shifts in biomass related to trends in climate [12, 13].

This low-frequency temporal variation in phytoplankton dynamics is especially interesting from the perspective of understanding the emergent properties of ecosystems, such as productivity and nutrient-cycling, over the decadal to centennial scales. In particular, understanding how the potentially very local dynamics of phytoplankton communities correlate over time throughout a large heterogeneous ecosystem may help us to understand the scale at which both ecosystem functions and phytoplankton dynamics operate. These local dynamics result from the diversity of phytoplankton communities with species having unique functional roles and evolutionary histories [14, 15], which respond to a broad array of abiotic and biotic variables [16, 17]. Low-frequency oscillations are often detected at coarser taxonomic scales, where functional roles are conserved [15], or because emergent properties from the underlying levels of biological organization appear.

The Baltic Sea is a large, brackish inland sea with a series of basins separated by shallow sills and latitudinal gradients in temperature and salinity. It naturally has relatively low biodiversity and a long history of human impacts including eutrophication, fishing, and contaminants [18]. The nations surrounding the Baltic Sea have supported a large-scale monitoring, including phytoplankton monitoring for water quality since the late 1970s [19]. Monitoring data have provided insights into the Baltic Sea’s long-term ecological dynamics including observed large-scale shifts in ecosystem states [e.g. 20, 21, 22] driven by a combination of both climatic and anthropogenic variables. Individual basins has been the primary scale of analysis and low-frequency climatic shifts are thought to synchronize the physical environment across basins [22].

The Baltic Sea summer phytoplankton bloom is crucial for pelagic production because high zooplankton abundances during this time period [23] increase transfer up the food web and, in addition, the sedimentation of the summer phytoplankton bloom provides an additional pulse of matter for benthic communities [24]. Despite substantial reductions in external nutrient loads in the past decade [25], internal recycling of nutrients continues to fuel eutrophication and the cyanobacterial dominance of the summer phytoplankton community. Monitoring data have been used to identify phytoplankton species [26] and class-specific trends and changes in community composition [27, 28]. Within Baltic Sea basins summer phytoplankton biomass contributed to analyses that identified the large-scale ecosystem shifts, which are in part in response to large scale climate forcing [22]. Analyses at the scale of the entire Baltic Sea would allow a better understanding of the degree to which large scale versus local environmental dynamics regulate phytoplankton dynamics. We investigated the potential for summer phytoplankton biomass to show common patterns of interannual variation, such as large-scale shifts, across the Baltic Sea by evaluating four alternative hypotheses:

1. Summer phytoplankton biomass exhibit common patterns of interannual variation across the Baltic Sea in response to large-scale climatic drivers.
2. Summer phytoplankton biomass exhibit patterns of interannual variation in response to local environmental dynamics and patterns will vary with the latitudinal gradient of temperature and salinity.
3. Summer phytoplankton biomass exhibit class-specific patterns of interannual variation due to their unique functional roles.
4. Summer phytoplankton biomass time series exhibit patterns of interannual variation that are regulated by both latitudinal environmental gradients and functional roles.

We evaluated the first two hypotheses using total summer phytoplankton biomass and all four hypotheses using summer biomass time series of the most common phytoplankton classes at stations across the Baltic Sea.

## Methods

### Data

#### Phytoplankton time series

**Fig 1:**
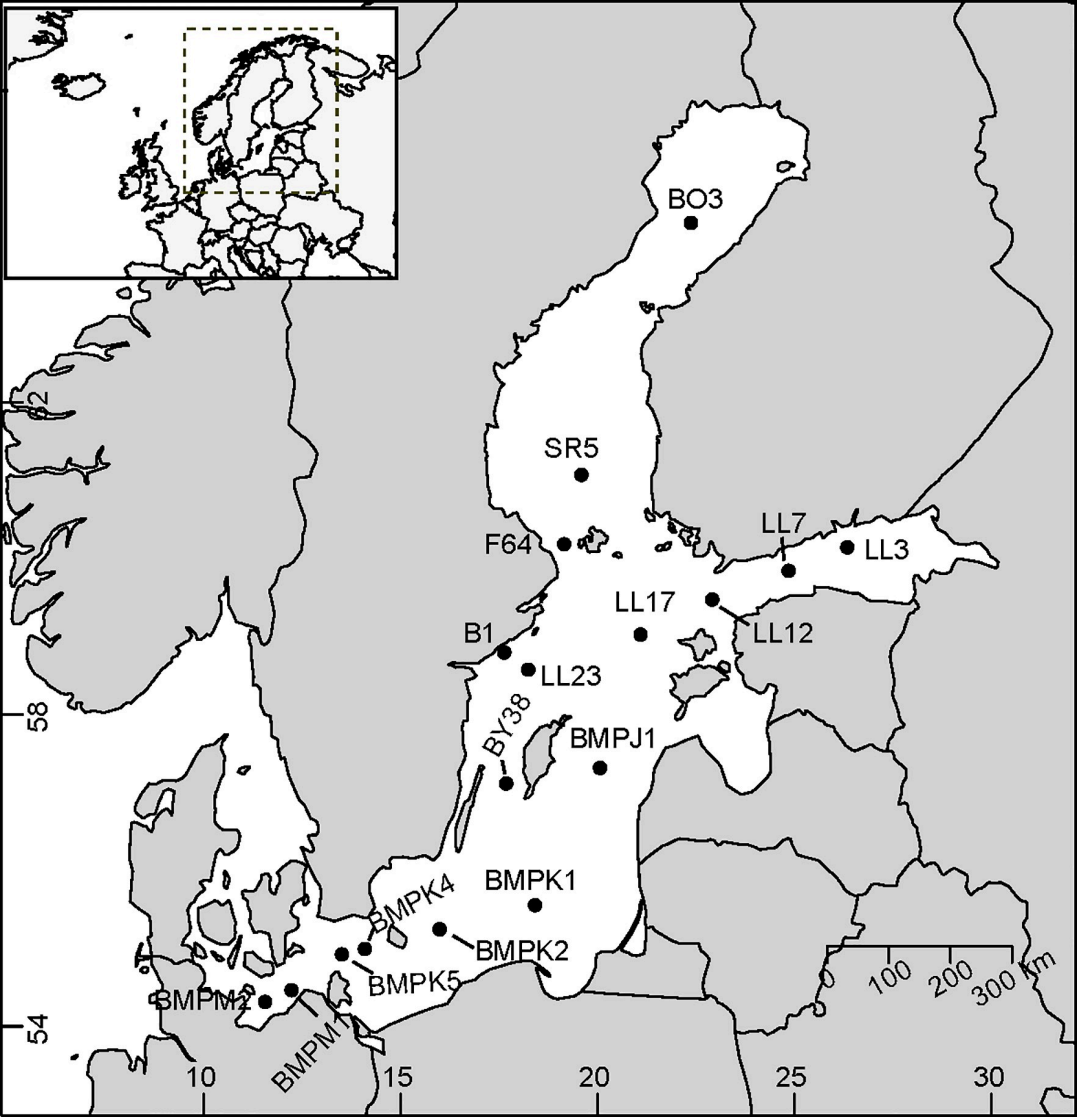
Map of the Baltic Sea study area and the location of phytoplankton sampling stations. Created using the package ‘maps’ in R [30].

Phytoplankton time series data for 17 stations across the Baltic Sea were obtained from numerous national monitoring programs (Fig 1, Table 1). These stations spanned 10 degrees in latitude (54° -64° N), a range of 3–10 in mean summer salinity and 14–18°C in mean summer temperature. Data were provided by the Leibniz-Institute for Baltic Sea Research [IOW, 27]; the Finnish Environment Institute [SYKE, 29]; and the Swedish Meteorological and Hydrological Institute (SMHI). Data were collected in July and August over the period 1979–2012 (34 years) and stations had a minimum of 20 years of data distributed throughout the time series. Stations were located in off-shore or exposed coastal habitats and phytoplankton were collected as 0-10m integrated water samples with the exception of one station (0-20m, site B1).

**Table 1:** Phytoplankton time series overview and sources. Stations are ordered from southwest to northeast.

Station	Latitude	Longitude	Phytoplankton Sample Depth	Years of Data	First, Last Year of Data	Min, Max Samples in July-August	Data Source	Data Contact/Weblink	Countries from which Monitoring Programs Contributed
BMP M2	54,32	11,55	0–10 m	30	1980, 2012	1, 4	IOW	N. Wasmund	DK, GDR, DE
BMP M1	54,47	12,22	0–10 m	30	1980, 2012	1, 4	IOW	N. Wasmund	DK, GDR, DE
BMP K5	54,93	13,50	0–10 m	28	1981, 2012	1, 4	IOW	N. Wasmund	DK, GDR, DE
BMP K4	55,00	14,08	0–10 m	31	1979, 2012	1, 5	IOW	N. Wasmund	DK, FI, GDR, DE, LT, USSR, SE
BMP K2	55,25	15,98	0–10 m	32	1979, 2012	1, 9	IOW	N. Wasmund	DK, FI, GDR, DE, LT, PL, USSR, SE
BMP K1	55,56	18,40	0–10 m	33	1979, 2012	1, 3	IOW	N. Wasmund	DK, FI, GDR, DE, LT, PL, USSR, SE
BY38	57,12	17,67	0–10 m	20	1979, 2012	1,1	SYKE	S. Lehtinen (-2008), https://www.emodnet-biology.eu (2009-)	FI
BMP J1	57,32	20,05	0–10 m	32	1979, 2012	1, 7	IOW	N. Wasmund	DK, EE, FI, GDR, DE, LV, LT, USSR, SE
LL23	58,58	18,23	0–10 m	23	1980, 2012	1,1	SYKE	S. Lehtinen (-2008), https://www.emodnet-biology.eu (2009-)	FI
B1	58,80	17,62	0–20 m	27	1984, 2011	4,5	SMHI	https://sharkweb.smhi.se	SE
LL17	59,03	21,08	0–10 m	25	1980, 2012	1,1	SYKE	S. Lehtinen (-2008), https://www.emodnet-biology.eu (2009-)	FI
LL12	59,48	22,90	0–10 m	26	1981, 2012	1,1	SYKE	S. Lehtinen (-2008), https://www.emodnet-biology.eu (2009-)	FI
LL7	59,85	24,84	0–10 m	30	1980, 2012	1,1	SYKE	S. Lehtinen (-2008), https://www.emodnet-biology.eu (2009-)	FI
LL3	60,15	26,33	0–10 m	26	1979, 2012	1,1	SYKE	S. Lehtinen (-2008), https://www.emodnet-biology.eu (2009-)	FI
F64	60,19	19,14	0–10 m	26	1979, 2012	1,1	SYKE	S. Lehtinen (-2008), https://www.emodnet-biology.eu (2009-)	FI
SR5	61,08	19,58	0–10 m	26	1979, 2012	1,1	SYKE	S. Lehtinen (-2008), https://www.emodnet-biology.eu (2009-)	FI
BO3	64,31	22,36	0–10 m	27	1979, 2012	1,1	SYKE	S. Lehtinen (-2008), https://www.emodnet-biology.eu (2009-)	FI

Source Abbreviations: IOW = Leibniz-Institute for Baltic Sea Research, SYKE = Finnish Environmental Institute

SMHI = Swedish Meteorological and Hydrological Institute

Country Abbreviations: DK = Denmark, EE = Estonia, FI = Finland, GDR = German Democratic Republic, DE = Germany, LV = Latvia

LT = Lithuania, PL = Poland, USSR = Soviet Union, SE = Sweden.

Phytoplankton samples were collected and analyzed according to the HELCOM COMBINE Baltic Sea monitoring guidelines [31]. Taxa were categorized by size class at the species or genus level and their biovolume was calculated from abundance and size-specific cell volumes according to Olenina et al. [32] and the HELCOM PEG (Phytoplankton Expert Group) Biovolume file (updated at: http://www.ices.dk/marine-data/Documents/ENV/PEG_BVOL.zip). The HELCOM PEG group ensures that phytoplankton data are comparable across monitoring programs. We obtained species level data from SMHI and SYKE while data obtained from IOW were already aggregated to orders from species level data to ensure compatibility among different data sources [see 27]. Data were reported as either biomass (IOW wet weight (mg m^-3^); SYKE wet weight (μg L^-1^)) or biovolume (SMHI μm^3^ L^-1^) and while we kept the data in the original units we will refer to biomass throughout the text for simplicity. In the SMHI dataset, rare species were not counted prior to 1992 and, to have a consistent dataset throughout the time series, we only included species in each sample contributing to approximately 90% of the biovolume in that sample.

The number of samples per year during this period varied between stations and within a station across years with a minimum of one and maximum of nine observations (Table 1). If multiple samples were taken on a single day at a station these were first averaged before calculating the July-August mean from all sampled dates. If multiple observations occurred within the July-August period they were generally at least two weeks apart, and these were all weighted equally in the calculation of the mean.

**Fig 2:**
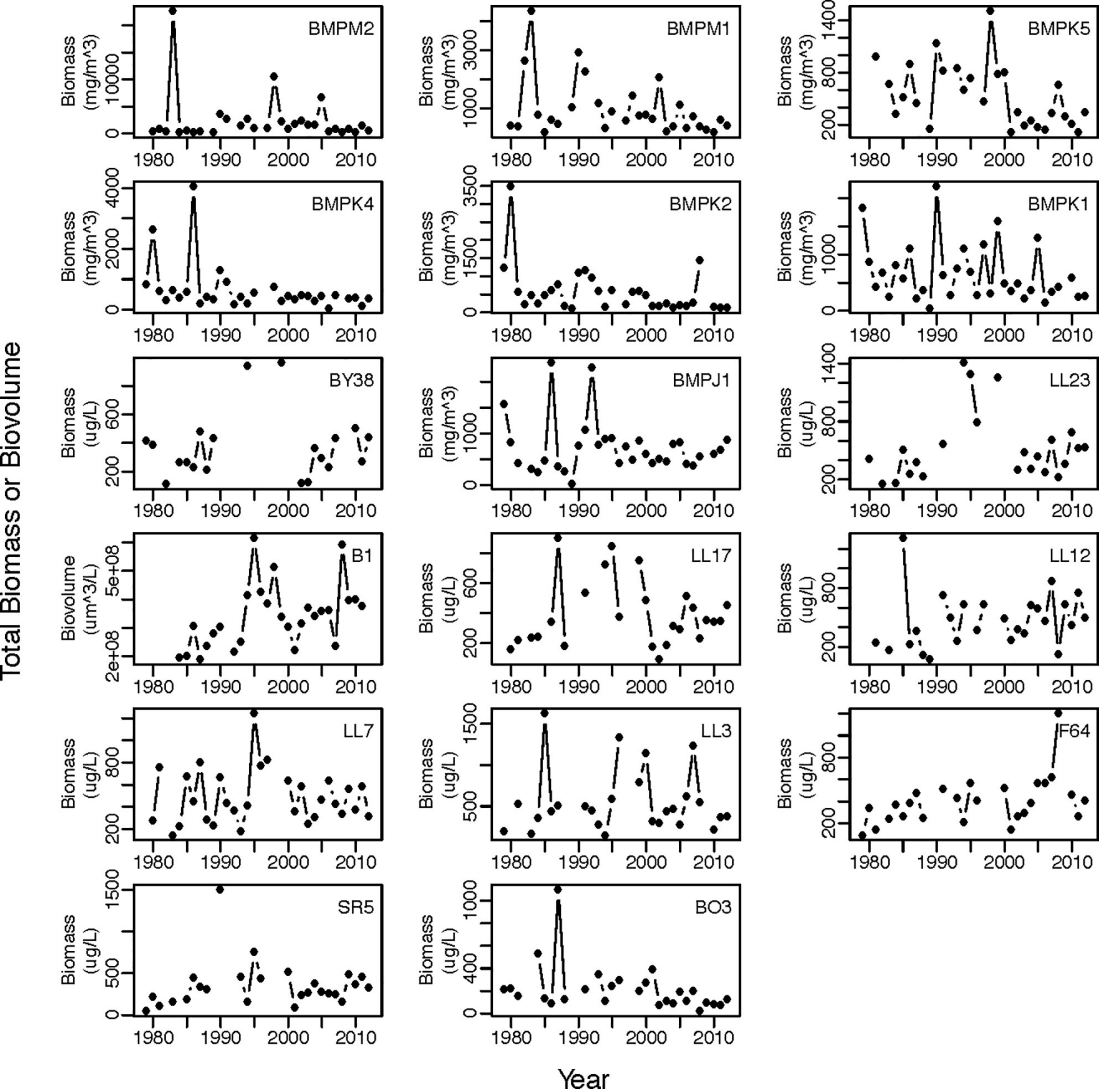
July-August mean total phytoplankton biomass or total phytoplankton biovolume for the different sampling stations in the Baltic Sea. Y-axis ranges and units differ among stations. Units are those as provided by the source monitoring program listed in Table 1 in the manuscript. Location of sampling stations are shown in Fig 1. Stations from top left to bottom right (by row) are ordered by geographic location (southwest to northeast).

July-August mean total biomass was calculated from all classes that were consistently measured throughout the time period at a given station (Fig 2, S1 Fig). For example, the large autotrophic ciliate, *Myrionecta rubra*, was included for the B1 station but excluded for all other stations. The class-level analyses focused on four main classes of phytoplankton (cyanobacteria, dinoflagellates, diatoms, and cryptophytes, S2 Fig, S3 Fig), which account for 47–94% of the July-August biomass across our stations and were always included in observations. We focused on mixo- and autotrophic phytoplankton except in the case of dinoflagellates where heterotrophs were not identified separately. For class-specific time series, if the July-August mean was zero, it was replaced by a random value between zero and half the minimum observed value [33] in the time series. For total biomass and class-level time series, data were natural log-transformed and standardized by z-scoring prior to statistical analysis.

#### Environmental time series

We used two measures of low-frequency climate-ocean variability which affect the abiotic conditions, especially stratification and mixing at large scales in Baltic Sea (S7 Fig). The North Atlantic Oscillation (NAO) principle component-based index values describe large scale climate variability over the North Atlantic [34]. Positive NAO anomalies are associated with warmer and wetter winter conditions in the Baltic region while negative anomalies are associated with colder and drier winters [35]. Baltic Sea circulation is also strongly affected by NAO phases resulting in differences in turbulent mixing and stratification among NAO phases [36]. We used mean winter NAO values (Dec-Mar) as these exhibit stronger inter-annual variability and regional forcing effects [37]. We also used the Baltic Sea Index [BSI, 36], which is correlated with the NAO but has been shown to have a stronger correlation with salt water inflows and climatic conditions in the Baltic region (Lehmann et al 2002). Updated monthly BSI values were provided by A. Lehmann (personal comm) and we assessed the explanatory power of both summer (July-August) and winter (Dec-Mar) mean index values. Positive BSI values are associated with wind conditions that enable saltwater inflows into the Baltic Sea from the North Sea while negative BSI values correspond to conditions that favor outflow from the Baltic.

Temperature and salinity time series were obtained for the majority of stations with phytoplankton data (see S1 Table for data source). SMHI environmental sampling was conducted simultaneously with phytoplankton sampling, while SYKE observations included both concurrent samples as well as additional environmental sampling. Data for other stations were obtained via the Baltic NEST system maintained by Stockholm University’s Baltic Sea Center and all July-August observations were used to calculate mean summer environmental conditions. The NEST system accesses data from numerous institutes and national monitoring programs and a full list of data sources is in S1 Table.

July-August mean monthly surface temperature and salinity values were calculated for each station (S7 Fig). For all stations we calculated the 0-10m average except for Station B1 (0-20m) to match the phytoplankton sampling depth range. We first took mean values for the depth interval by day and then over the July-August period. Depth intervals differed by station and sampling date but as we focus on surface water above the thermocline, this effect should be minimal. The number of samples in a month differed within and among stations with a minimum of one and a maximum of nine observations.

### Statistical analyses

#### Phytoplankton time series trends

We evaluated our hypotheses regarding common patterns of interannual variation among phytoplankton time series across the Baltic Sea using Dynamic Factor Analysis [DFA, 38, 39]. DFA can be considered a principle component analysis (PCA) for time series where the time-ordered nature of the data is explicitly considered [39]. In analyses where we expect time series to be correlated (e.g. populations responding to the same environmental driver), DFA is used as a dimension-reduction technique (like PCA) to describe underlying common patterns [called "common trends," 38] in multivariate response variables (the time series) and determine effects of explanatory variables on these time series [e.g. 40]. The relationship of individual time series to estimated common trend(s) of the data can be assessed using the loadings of the individual time series [39].

We use the notation of Holmes et al. [41] to describe the mathematical relationship between estimated common trends and the observed time series. DFA is a form of state-space model where an *m* number of unknown common trends (*X*) are modeled as random walks in the state model (Eq 1). When multiple common trends are found, the order of the trends does not reflect relative importance or explanatory power. The relationship of the observed time series (*Y*, *e*.*g*. *phytoplankton time series*) to the trends (*X*) is estimated in a *Z* matrix in the observation model (Eq 2) and are described by the loadings. The absolute value of a loading reflects the relative importance of an estimated trend in explaining variance in an observed time series and the sign indicates whether or not the time series is positively or negatively related to the estimated common trend. We evaluate only factor loadings with an absolute value greater than 0.2 [39].

The level parameter, *a*, in Eq 2 was set to zero because data were z-scored [see 42]. The process error, *w*_*t*_, is multivariate normally distributed (MVN) and we set the state variance-covariance matrix, *Q*, to identity [39]. The observation error, *v*_*t*_, was also multivariate normally distributed and estimated within the variance-covariance matrix, *R*. The parameters of the variance-covariance matrix are estimated and different configurations of the parameters allow for comparisons of alternative hypotheses regarding observation error (e.g. whether the variance is drawn from the same or different normal distributions with a mean equal to zero and a variance equal to the estimated parameter). Including covariates within the model structure removes the effect of known important drivers [42] so that the remaining common trends explain shared responses to unknown drivers. If covariates (e.g. environmental variables) are included in the observation model, *d* is a vector of the covariate at time *t* and *D* is the effect of the covariate on the observations, *Y*. Covariate time series (*d*) cannot have missing data. The model structure can estimate the same effect of *d* on all *Y* time series (estimate one parameter in the *D* matrix) or a unique effect of *d* for each *Y* time series (estimate many parameters in the *D* matrix).

$$X_{t}=X_{t-1}+w_{t},\;w_{t}∼MVN\;(O,Q)$$ (Eq 1)

$$Y_{t}={ZX}_{t}+a+{Dd}_{t}+v_{t},\;v_{t}∼MVN\;(O,R)$$ (Eq 2)

We used separate DFA analyses biomass anomalies for total and class biomass time series to test our hypotheses regarding the presence of underlying common trends. Akaike’s Information Criteria for small sample sizes [AICc, 43] as suggested by Zuur et al. [39] was used to evaluate support from the data for alternative hypotheses. Relative support for alternative hypotheses was assessed by computing the AICc differences (dAICc) [dAICc = AICc—AICc min, 43] where differences of 2 or less show stronger support for those models while differences greater than 10 show very little support relative to the model with the lowest AICc.

#### Total biomass model structures

To evaluate two alternative hypotheses, whether there are common interannual patterns in phytoplankton biomass to large-scale climate forcing or whether those patterns reflect local environmental conditions across latitude, we tested different model structures using 17 time series (1 per station) of total biomass anomalies across the Baltic Sea. We used AICc to find the model with the most support for between one and four common trends. Using the variance-covariance matrix, we also tested different parameter structures to see if the variance unexplained by common trends was similar or different among time series. The parameter structures of the variance-covariance matrix (R-matrix) included: (1) shared variance parameter among time series with no covariance (“diagonal and equal”); (2) different variance parameters by time series with no covariance (“diagonal and unequal”); or (3) shared variance parameter among time series and shared covariance parameter (“equal variance covariance”).

To directly estimate the effect of regional climate indices on phytoplankton biomass we included them as covariates in alternative models. The regional climate indices (Winter NAO, Winter BSI, July-August BSI) had no missing values and were included individually in the model structures described above for one or two common trends. Finally, to test for no common trend but a direct effect of the regional climate indices on phytoplankton biomass we fit models containing each regional climate covariate individually without a trend parameter (i.e. multiple regression). For all models that included covariates, we compared a shared parameter among phytoplankton time series (e.g. Winter NAO has the same effect on all time series) and unique parameter among phytoplankton time series (e.g. Winter NAO has different effects on individual time series).

#### Class biomass model structures

To evaluate all four of our hypotheses regarding patterns in interannual variation among phytoplankton biomass time series, we conducted a DFA analysis using four phytoplankton class time series (cyanobacteria, dinoflagellates, diatoms, and cryptophytes) from each of the 17 stations (68 times series in total). We assessed the AICc support for models including from one to four common trends among the time series (additional analyses indicated no support for a greater number of trends) with similar AICc thresholds as in the total biomass model (see above). The relationship of estimated common trends across the environmental gradient to taxonomic identity was assessed using the patterns of the trend loadings (e.g. time series of the same class or Baltic Sea basin had the same loading, positive or negative for the same trend). We also evaluated whether unexplained variance in the trends was structured by taxonomic identity (class) or geographic location (station), and if it was unique or common across all stations and classes to further understand the geographic and taxonomic effects on the time series. This was done by assessing alternative parameter structures for the R variance-covariance matrix. These included: (1) common variance parameter across all time series; (2) unique variance parameters for all time series; (3) common variance parameter among time series from the same station; and (4) common variance parameter among time series of the same class.

We expected that regional climate indices exhibit low-frequency variation that should primarily be related to the common trends and, due to the large number of time series, we did not include environmental covariates directly in the model because it would have required estimating a large number of parameters. Instead, we did a post-hoc exploration of the correlation between the regional environmental time series (Winter NAO, Winter BSI, July-August BSI) to the trends from the AICc selected model.

Local environmental variables, station temperature and salinity, which could drive basin-specific patterns had missing data and could not be included in the DFA model as covariates. After the most parsimonious model was selected, we conducted a post-hoc correlation analysis between the residual variance in a given time series and its station environmental variables. We calculated Spearman’s rank correlation coefficients between either trend values or time series residuals and the environmental time series. For the correlation between trends and regional climate variables, the degrees of freedom were adjusted to account for lag-1 autocorrelation within the time series using the modified Chelton method [equation 7 in 44].

We conducted a follow-up analysis to assess the sensitivity of the model selection to extreme values in the data. We assessed the effects of two years, 1990 and 2008, in which cryptophytes were not observed (large negative anomaly) at a large number of stations in the southern (1990) and northern (2008) Baltic Sea. For these two years, we replaced observed values with NA in all time series (time series were re-z-scored to reflect the removal of data) and we again conducted the DFA model selection and assessed the most parsimonious model’s trends and loadings.

#### Analysis software and protocol

All statistical analyses were conducted in R [45]. Packages used include reshape2 [46], MARSS [41, 47], and TMB [48]. We performed initial DFA model fitting using TMB (DFA code written by T.J. Cline), which substantially improved DFA model fitting speed using BGFS optimization. We used 20 randomized starts to ensure that likelihood values were not trapped in local minima. We also ran final models to convergence in the MARSS package using the EM algorithm for optimization initiating the model with randomized starting conditions. From the final models we evaluated residuals for homogeneity, normality, and autocorrelation. We assessed goodness of fit for AICc selected models by calculating r^2^ values for each time series and across all time series.

## Results

### Total biomass analysis

Variation of total phytoplankton in July-August was poorly explained by all DFA model structures, suggesting that there is no support for the hypothesis that mean summer phytoplankton biomass time series across the Baltic Sea exhibited common interannual patterns over the past three decades (S4 Fig). Regional climate variables were not strong predictors of total biomass variation (S2 Table). Given the models explored, model selection supported the inclusion of one trend (see S2 Table). The AICc value for the model with a trend and no covariates was only slightly better than including one climate covariate with a shared coefficient. The addition of this climate variable also did not improve the goodness of fit (no covariate, overall r^2^ = 0.15; with covariate, overall r^2^ = 0.15). All models with a dAICc under 10 showed support for an error structure with a shared variance parameter (top model, 0.85) and covariance parameter (top model, 0.23). The loadings by each time series onto the single trend were weak overall with only five phytoplankton time series having loadings greater than |0.2| (S4 Fig), suggesting that about a third of the time series were related to the trend. The open coastal station in the northern Baltic Proper, B1, was most strongly related to the estimated trend.

Model residual plots indicated that residuals were normal and homogeneous. When evaluating autocorrelation of the residuals, we focused on lag-1 autocorrelation, and there was a significant lag-1 autocorrelation for station LL17 (autocorrelation value = 0.37).

### Class biomass analysis

#### Common trends

The overall hypothesis that there are common interannual patterns in phytoplankton taxonomic class biomass was supported by the DFA analysis with some important caveats, which are discussed below. There was strong AICc support for a model structure that included three estimated common trends for phytoplankton biomass class time series across the Baltic Sea and unique variance parameters by phytoplankton class (Table 2). This model structure had a 15 AICc unit difference from the next best model. Across all time series this model explained 37% of the variance in the data, however, explanatory power varied strongly among time series (r^2^ range 0.02–0.84, Table 3, see S6 Fig for observed and predicted time series). Observed cryptophyte time series had substantially higher r^2^ values (range 0.32–0.84) than the time series of the other phytoplankton classes. The common trends were related to both functional group (cryptophytes) as well as the geographic location of the stations (north or south). Among observed cyanobacteria time series, explanatory power ranged from 1% (Station K1) to 55% (Station BO3). There was a similar range for dinoflagellates (2% to 54%) and diatoms (7% to 53%). Although this model supported the hypothesis that variance differs among classes (e.g. a unique variance, R, is estimated for each class), the major difference among the estimated parameters was between cryptophytes (lower variance, R = 0.33) and the other three classes (higher variance, cyanobacteria = 0.69, dinoflagellates = 0.71, diatoms = 0.79).

**Table 2:** DFA model selection for class time series model using T. Cline’s DFA with the TMB package. Each model structure has been initialized with random start values (20 initiations) to ensure estimates not stuck in local minima. Models were fit to 68 phytoplankton biomass time series (class x station). R-structure is the variance-covariance matrix structure. The AICc and dAICc values are for the lowest of the iterations for a given model structure.

R structure	Trends	Parameters	N	AICc	dAICc
Class	3	205	1888	5070,75	0,00
Class	4	270	1888	5086,43	15,67
Class	2	139	1888	5090,62	19,87
Diagonal and Equal	3	202	1888	5128,50	57,75
Diagonal and Equal	2	136	1888	5135,01	64,26
Class	1	72	1888	5142,19	71,44
Diagonal and Equal	4	267	1888	5151,96	81,20
Station	2	152	1888	5162,71	91,95
Station	3	218	1888	5165,08	94,33
Diagonal and Equal	1	69	1888	5170,97	100,22
Diagonal and unequal	3	269	1888	5177,43	106,67
Diagonal and unequal	4	334	1888	5190,29	119,54
Station	4	283	1888	5193,64	122,89
Station	1	85	1888	5202,19	131,44
Diagonal and unequal	2	203	1888	5202,70	131,95
Diagonal and unequal	1	136	1888	5255,51	184,76

**Table 3:** Goodness of fit (r^2^) for AICc selected model. The r^2^ is shown for each time series and overall across all time series.

Time Series			Time Series		
Station	Class	r^2^	Station	Class	r^2^
BMPM2	Cryptophytes	0,65	B1	Cryptophytes	0,32
BMPM2	Diatoms	0,24	B1	Diatoms	0,12
BMPM2	Dinoflagellates	0,49	B1	Dinoflagellates	0,11
BMPM2	Cyanobacteria	0,20	B1	Cyanobacteria	0,45
BMPM1	Cryptophytes	0,62	LL17	Cryptophytes	0,78
BMPM1	Diatoms	0,26	LL17	Diatoms	0,16
BMPM1	Dinoflagellates	0,14	LL17	Dinoflagellates	0,45
BMPM1	Cyanobacteria	0,27	LL17	Cyanobacteria	0,53
BMPK5	Cryptophytes	0,84	LL12	Cryptophytes	0,72
BMPK5	Diatoms	0,29	LL12	Diatoms	0,13
BMPK5	Dinoflagellates	0,36	LL12	Dinoflagellates	0,54
BMPK5	Cyanobacteria	0,25	LL12	Cyanobacteria	0,18
BMPK4	Cryptophytes	0,75	LL7	Cryptophytes	0,79
BMPK4	Diatoms	0,08	LL7	Diatoms	0,25
BMPK4	Dinoflagellates	0,33	LL7	Dinoflagellates	0,03
BMPK4	Cyanobacteria	0,38	LL7	Cyanobacteria	0,28
BMPK2	Cryptophytes	0,74	LL3	Cryptophytes	0,64
BMPK2	Diatoms	0,10	LL3	Diatoms	0,53
BMPK2	Dinoflagellates	0,14	LL3	Dinoflagellates	0,02
BMPK2	Cyanobacteria	0,11	LL3	Cyanobacteria	0,16
BMPK1	Cryptophytes	0,62	F64	Cryptophytes	0,83
BMPK1	Diatoms	0,19	F64	Diatoms	0,23
BMPK1	Dinoflagellates	0,39	F64	Dinoflagellates	0,17
BMPK1	Cyanobacteria	0,13	F64	Cyanobacteria	0,41
BY38	Cryptophytes	0,80	SR5	Cryptophytes	0,61
BY38	Diatoms	0,14	SR5	Diatoms	0,11
BY38	Dinoflagellates	0,19	SR5	Dinoflagellates	0,30
BY38	Cyanobacteria	0,53	SR5	Cyanobacteria	0,40
BMPJ1	Cryptophytes	0,57	BO3	Cryptophytes	0,82
BMPJ1	Diatoms	0,12	BO3	Diatoms	0,24
BMPJ1	Dinoflagellates	0,38	BO3	Dinoflagellates	0,20
BMPJ1	Cyanobacteria	0,18	BO3	Cyanobacteria	0,56
LL23	Cryptophytes	0,69			
LL23	Diatoms	0,09			
LL23	Dinoflagellates	0,50			
LL23	Cyanobacteria	0,43			
	**Overall**	**0,37**			

**Fig 3:**
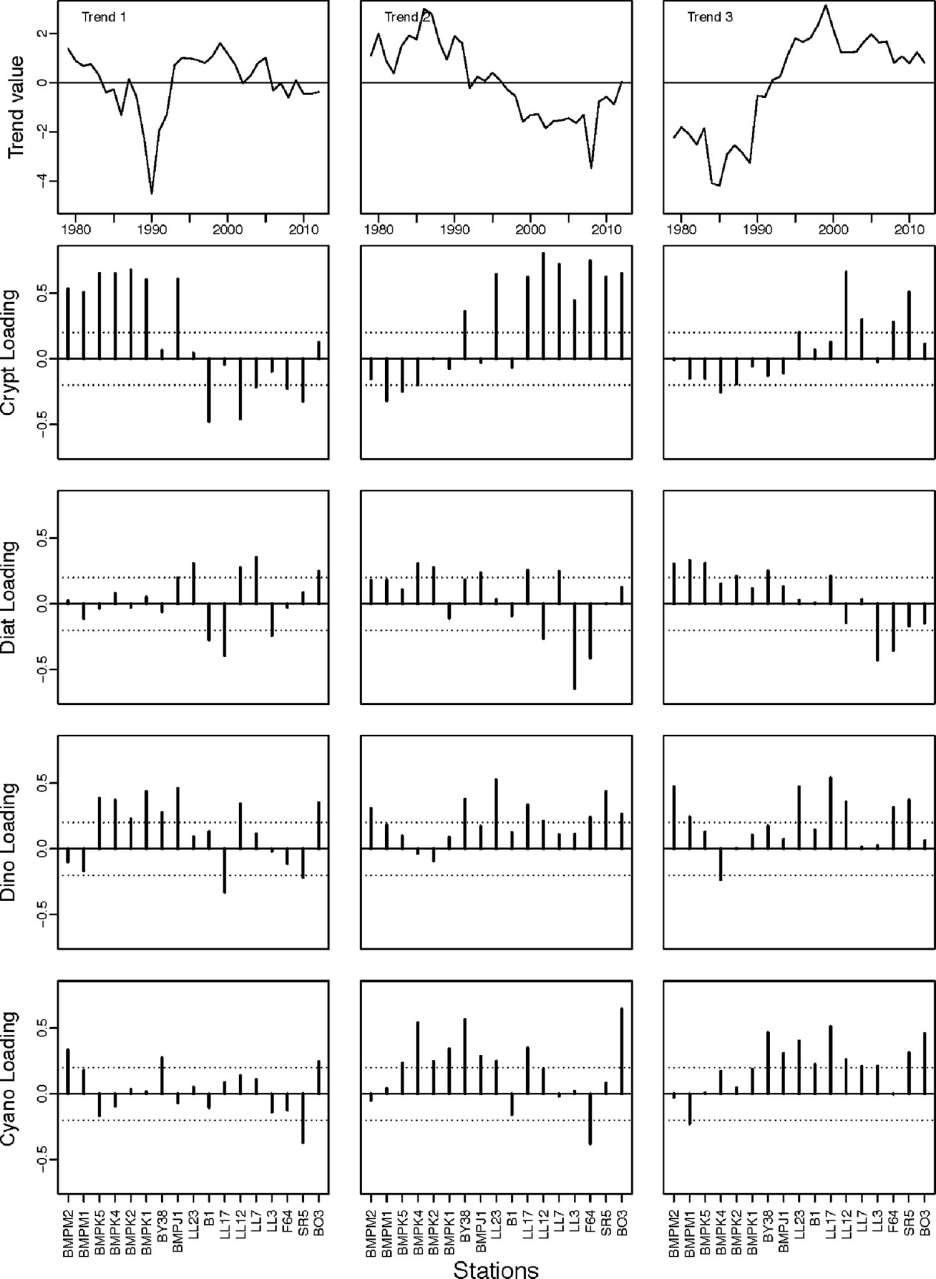
Shared trends and loadings from the most parsimonious model. That trend number does not reflect importance or explanatory power. Trend values are unitless. Loadings are the relationships of each time series to each trend; stations are ordered (left to right) from southwest to northeast.

Estimated common trends 1 and 2 were both associated by strong loadings from the observed cryptophyte time series (Fig 3). Trend 1 displayed small oscillations above or at the long-term mean except for a sharp decline in 1990. Observed cryptophyte time series from the southern Baltic Sea (all BMPM stations) showed a strong positive relationship to trend 1, while dinoflagellates from these stations showed a similar but weaker response. In contrast, trend 2 was marked by an above average period in the first half of the time series and a below average period in the second half including a steep decrease in 2008. The 2008 decline was strongly related to an absence of cryptophytes in most of the northern Baltic Sea stations. Across the Baltic Sea, some observed cyanobacteria time series across the Baltic Sea showed a positive association with this trend characterizing a shift from above to below average biomass with no consistent regional pattern. Estimated trend 3 was characterized by a below average decade followed by two above average decades (Fig 3). Primarily dinoflagellate and cyanobacteria time series from central and northern Baltic stations are positively related to trend 3 as well as two cryptophyte time series.

In general, residuals for all time series were normally distributed and did not show any changes in variance across fitted values. Inspection of residuals across time found that nine out of the 68 time series had significant lag-1 auto-correlations, including all four time series from the station LL23. This is a small proportion of the time series but it indicates that we should interpret results for these time series with caution.

#### Environmental correlations

**Fig 4:**
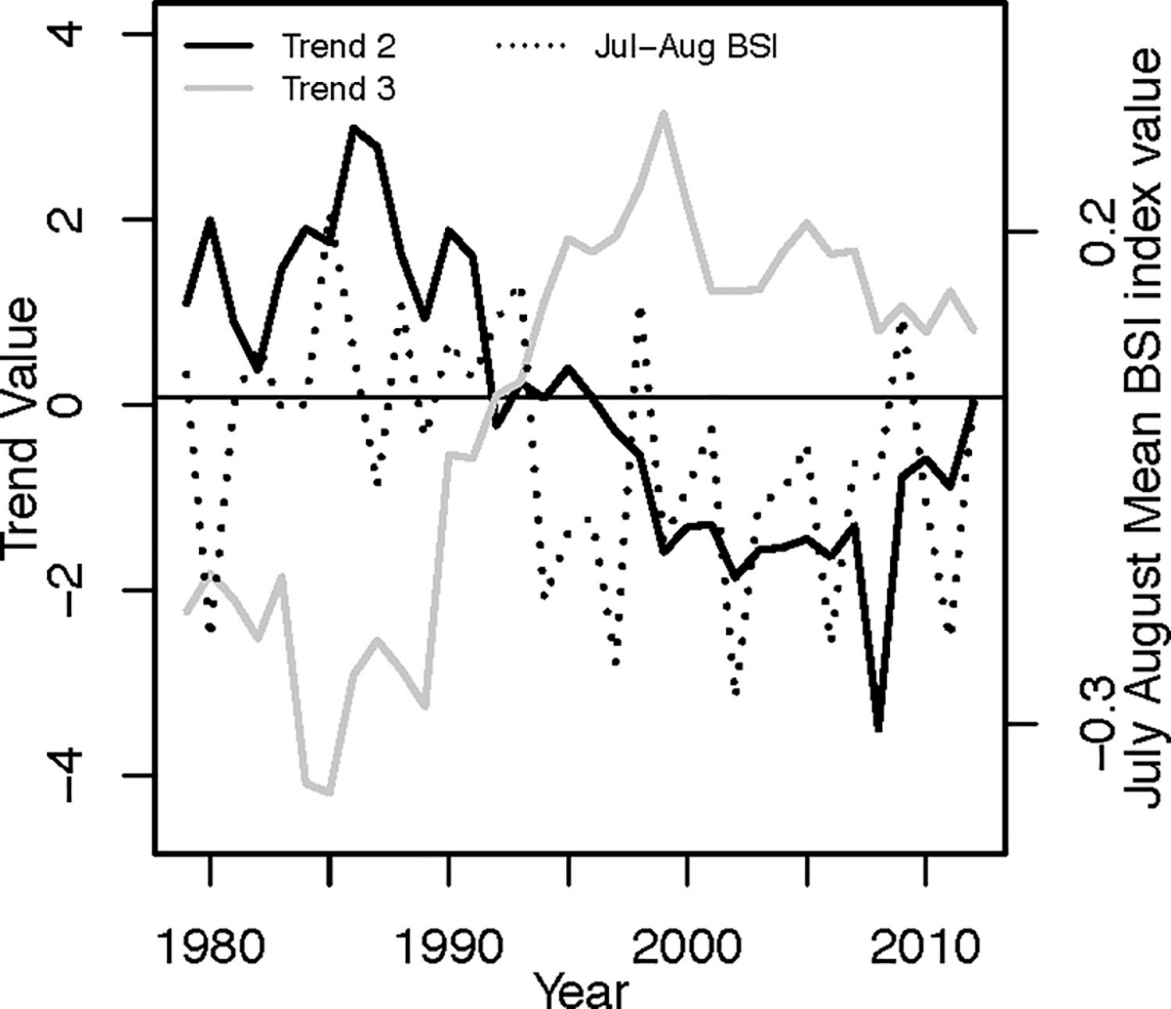
Trend and Baltic Sea Index values. July-August Mean Baltic Sea Index values and the values of trends that are correlated with it.

Trend 2 (Spearman’s r = 0.44, p < 0.05) and trend 3 (Spearman’s r = -0.49, p < 0.05) were significantly correlated with the July-August BSI index values (Fig 4, S3 Table). A primarily positive July-August BSI phase prior to 1995 was positively related to Trend 2 and inversely related to Trend 3 and a similar pattern persisted after the BSI switched to a below average phase around 1995.

In an alternative approach, we explored the ability of local environmental conditions (surface water temperature and salinity) to explain additional variation in individual phytoplankton time series. Residuals for each phytoplankton class time series from the AICc selected model fit were correlated with local environmental time series of temperature and salinity. For the 15 stations with environmental data (this excluded BMPM2 and BMPK5), we found 10 significant correlations with the residuals of a class time series and environmental conditions (S4 Table). These time series came from only 6 of the stations, including three of four Station B1 time series showing a significant relationship with surface water temperature. Salinity explained significant variation in the residuals in 6 time series from a wide geographic range including the southwestern, central, and northern Baltic Sea. There were no obvious groupings between the time series attributes (class or geographic location) and the variables found to be significant. Time series showing significant residual relationships to the environment also differed strongly in the extent to which their biomass variation was well explained by the original model (r^2^ 0.02–0.57).

#### Model selection and trend sensitivity

Model selection was sensitive to the influence of the large negative cryptophyte anomalies (e.g. biomass of zero) in 1990 and 2008. When data for those years were replaced by NA in all time series, a model structure of two common trends was selected and there continued to be support for the variance-covariance matrix with variance parameters by phytoplankton class. The first trend oscillated around the mean (S6 Fig) and was most strongly associated with the observed south and central Baltic Sea cryptophyte time series and a limited number of diatom and dinoflagellate time series. In contrast, trend 2 displayed a period of above average values followed by a switch to below average values in 1995 (S6 Fig). However, very few time series had loadings above |0.2| onto trend 2, indicating it was not particularly informative for explaining variation in the observed time series. The r^2^ was 0.25 with a range of 0.01–0.79 across time series indicating that there was highly variable explanatory power.

## Discussion

Baltic Sea summer phytoplankton biomass patterns show some common decadal trends, which may relate to regional climate indices but the evidence is relatively weak and overall suggests the importance of local environmental conditions. We found no strong support for the hypothesis that total summer phytoplankton biomass would show interannual patterns driven by regional climate indices. There was also little evidence, however, that stations within basins had common trends. For the most common phytoplankton classes (cyanobacteria, diatoms, dinoflagellates, and cryptophytes) that regularly contribute to summer biomass, we do find some evidence that both class identity and geography explain their temporal variance resulting in common trends. The common trends are most closely associated with cryptophyte time series across the Baltic Sea and are particularly sensitive to the latitudinal pattern of cryptophyte anomalies in two years. Without these years influencing the analysis, there is less evidence for common trends among class time series and stations. However, declines in cyanobacteria at some southern Baltic Sea stations were present in both analyses.

It was surprising that no overall shift in ecosystem state, described by common trends, was detected for total biomass and with limited evidence for class biomass at stations across the Baltic Sea even though one or more large-scale ecosystem shifts have been documented in the Baltic Sea in the last half-century [e.g. 20, 21, 22]. Although phytoplankton dynamics may fluctuate unpredictably, it is expected that greater coherence and predictability will occur at higher levels of aggregation [49]. Moreover, Olli et al. [50] estimated the spatial and temporal autocorrelation in phytoplankton monitoring samples (June-August) and found support for moderate levels of spatial autocorrelation up to 400 km in the Baltic Sea. Similarly, selected phytoplankton species across the Baltic Sea showed strong spatial synchrony [26]. In light of this finding, we expected to see greater support for common interannual patterns of variation at least for stations within the same basin and especially those in close proximity, known to have the same environmental conditions [see comment on BMPM1, BMPM2 as well as BMPK4, BMPK5 in 27]. In previous analyses, summer chlorophyll *a* and/or summer phytoplankton biomass were linked to ecosystem shifts in some basins [22] and basin-specific studies have documented long-term trends in the biomass of some classes [27, 29]. In studies considering entire ecosystem shifts, however, summer phytoplankton showed weaker responses in many cases than other trophic levels or stronger responses were seen using chlorophyll *a* than class biomass [21, 22]. We discuss how our results are similar or different to previous studies for specific classes below as well as broader ecological and data considerations when interpreting our results. Our model structure allowed for the evaluation of alternative hypotheses for how interannual patterns of summer phytoplankton variation may be organized across the Baltic Sea (e.g. within basins, across basins, or within or across classes) without having to compare trends or parameters between models developed with different subsets of the data. Overall, the limited support for common interannual patterns in biomass indicates that emergent properties at the base of the food web should be characterized at a relatively local scale although some classes may respond to common environmental conditions across several basins (e.g. cryptophytes in the northern Baltic Sea).

### Patterns by phytoplankton class

Cryptophyte time series were the most influential in generating common trends among the 68 class time series, yet their contribution to summer biomass is on average relatively small (mean percent of total biomass 5–21% across our stations). Interestingly, the strong negative anomalies, which strongly influenced the detected trends, did not occur in the same Baltic Sea basins. The 1990 negative anomaly was primarily in the southwestern and southern Baltic Sea, while the 2008 anomaly occurred across the north-central and northern Baltic Sea. Perhaps due to these key differences in the timing of negative anomalies, the southern Baltic cryptophyte time series primarily were related to an estimated trend showing oscillations around the mean with the exception of the 1990 anomaly. On the other hand, north-central and northern stations were most strongly related to an estimated trend showing shift from above to below average biomass over time. This is consistent with Suikkanen et al. [29] who found decreasing trends for cryptophytes at the basin scale (for northern basins). The Baltic Sea has warmed rapidly since the early 1980s [35], in particular in the north, and cryptophytes are thought to prefer cooler temperatures [29].

Cyanobacteria play an important role in summer communities in the Baltic Sea. Several key species bring new sources of nitrogen to the Baltic Sea through nitrogen fixation [51, 52] and their large, dense blooms are a symptom of and contributor to ongoing eutrophication [53]. In the 17 stations used in this study, cyanobacteria contribute between 3–61% on average to the total summer biomass (if the northernmost site, BO3 is excluded, the range among stations is 20–61%). Satellite studies suggest that cyanobacteria in the Baltic Sea show decadal scale oscillations in spatial extent [54] and within these decadal scale patterns oscillation periods in intensity of about three years, which could not be explained by any abiotic factor [55]. On the other hand, studies of individual stations suggested both biomass declines [27] and increases [29], which may reflect differences in methodology (i.e. limited number of monitoring samples) as well as specific dynamics at the station. Given that we used the same data as these station-specific studies, it is not surprising that times series from two southern Baltic Sea stations were strongly associated with the above to below average shifts in trend 2, while times series from several central and northern stations were associated with the inverse pattern of trend 3. This common shift to below average biomass at some southern and central stations was retained even in analyses that removed the influential cryptophyte years. Cyanobacteria are known to prefer warmer temperatures but significant residual variation was not explained by local surface temperatures and it may be that the effects of ongoing warming [35] are seen in changes in phenology [e.g. 54] rather than biomass variation.

Neither observed dinoflagellate nor diatom time series showed clear relationships to the common trends. The dinoflagellate loadings showed little spatial coherence because positive loadings on all three trends were estimated across the north-south gradient in the Baltic Sea. Diatoms also showed little similarity in loading response and had overall weak loadings such that little variation in the observed data were explained by the common trends. Summer diatom biomass, in particular, has shown little connection to larger scale ecosystem shifts in most basins in previous studies [21, 22]. Dinoflagellates and diatoms account for four of the six most diverse genera [50] and the dynamics of dinoflagellates may not be well-represented at the class level and instead may be more synchronous if grouped more narrowly by traits of species.

### Effect of environmental variables

The summer BSI index was correlated with two common class trends (cryptophytes and cynaobacteria), both showing shifts in mean state around the mid-1990s. However, we do not have a strong mechanistic explanation for the relationship of the summer BSI index to the main drivers of the common trends (anomalously low cryptophyte years and below average cyanobacteria biomass in the southern Baltic Sea). Local environmental variables (water temperature and salinity) are also able to explain additional residual variation in 15% of observed time series that had local environmental data available. Nutrient availability, especially nitrogen in summer, may also drive phytoplankton biomass and production. We were not able to include nutrient data, however, because inorganic nutrient concentrations during the summer in the Baltic Sea are low and nitrogen availability in surface waters is primarily due to recycling and fixation by cyanobacteria [56].

### Challenges of working with complex data

The ability of our analyses to detect common interannual patterns could be affected by how representative both the stations and the samples are of phytoplankton biomass throughout the Baltic Sea. Monitoring stations are typically selected because they reflect the common characteristics of their basin [57]. Olli et al. (2013) determined biomass autocorrelations are observed up to 400 km, suggesting that stations should be representative of basin conditions. In addition, all the stations included in this study were offshore stations, with the exception of B1. The offshore environment is expected to be more homogenous over large spatial scales unlike coastal stations, which are more directly affected by the complex topography and inputs from terrestrial systems. Therefore, offshore stations are more likely to be representative of a large area. Station B1, although a coastal station, is relatively exposed and likely to be less affected by local processes than most coastal stations.

While stations are expected to reflect the conditions present across large spatial areas, phytoplankton populations are known to exhibit high frequency variability [9]. Some stations (primarily in the northern Baltic) had only one summer sample in most or all years, which may have more poorly characterized mean summer conditions than stations that had multiple samples taken each year. The potentially random nature of these samples could have made it difficult for northern Baltic Sea stations to exhibit common trends. An analysis on a smaller region of the Baltic Sea found that sites have the greatest similarity during high biomass periods [e.g. summer, 58]. Moreover, several of these northern Baltic Sea stations with only a single summer sample were used to detect significant trends for some taxa in other studies [26, 29, 59, 60]. Finally, the cryptophyte anomalies which occurred in different years at northern (1 summer sample) and southern (multiple summer samples) stations were equally detectable and wide-spread.

While we chose to analyze the data at the class level to account for key functional groups, there are some functional differences among taxa within these classes. Using finer scale groupings may have increased the likelihood of observing within functional group shared trends across space (especially for dinoflagellates and diatoms, see above). However, we elected not to pursue this approach due to the substantial increase in the total number of times series that would be modelled.

Summer bloom dynamics may be less conserved across time and space unlike spring bloom dynamics that can be synchronized at larger spatial scales due to large-scale environmental drivers (ice melt, changing light conditions) and the low-frequency climate variability (NAO) that influences ice cover and temperature. Both summer and spring dynamics have been demonstrated to be distinct and conserved, but summer dynamics were much less synchronized across locations [61], while interactions within the phytoplankton community also differ widely among locations [62]. Biological interactions as well as local environmental conditions may ultimately affect interannual patterns of variation more than large-scale ecosystem shifts.

Biomass observations are affected by grazing pressure, which in turn can influence the ability to detect common trends. This would likely have the greatest effect on cryptophytes and diatoms which are less common in summer communities but are high-quality food items for zooplankton [63] and may be found more frequently as diet items than expected based on their concentration in the water column [64]. Grazing pressure could result in common interannual patterns in phytoplankton biomass. In the central Baltic Sea, for example, summer zooplankton biomass, and in turn trophic cascades, strongly predicted phytoplankton variation at the basin scale [65], but recent analyses of zooplankton monitoring data suggest high variability in zooplankton community dynamics among basins [66].

An advantage of the DFA framework is that it estimates trends using a random walk, which allows for autocorrelation and can generate many different patterns of interannual variation. Low-frequency oscillations, such as the observed shifts in ecosystem state in the Baltic Sea, should be well-described. However, if biomass time series have limited autocorrelation among years or the dynamics are highly stochastic among years, this method may not describe the dynamics as well. However, it is important to use a time-dimension reduction method that is explicit about the time-order nature of time series data which traditional PCA are unable to account for [67]. In addition, the time series used in this analysis vary in the number of summer observations both among time series as well as within time series among years. This may limit our ability to detect trends, especially given the stochastic nature of phytoplankton dynamics within seasons.

## Conclusion

The Baltic Sea summer phytoplankton community plays an important role in biogeochemical cycling, especially through the fixation of atmospheric nitrogen, and in the transfer of energy through the food web via zooplankton. Studies characterizing this summer community have focused at different levels of taxonomic resolution and spatial resolution and, as a result, documented a wide range of temporal patterns. Here we show that a few strong anomalies result in support for a model of shared trends in class biomass across the Baltic Sea but that without these anomalies the support is much reduced. Therefore, low-frequency dynamics in summer Baltic Sea biomass, if present, are not responding to a common environmental driver. This implies that we need to be cautious about extrapolating in space regarding long-term patterns in the phytoplankton community but also acknowledge that the emergent ecosystem dynamics maybe bear little resemblance to the localized dynamics of communities. In particular, taxa higher in the food web will integrate over a larger spatial scale, smoothing out the localized stochasticity of phytoplankton dynamics. Identifying the scales at which phytoplankton dynamics act in specific ecosystems using multiple analytical frameworks is crucial to evaluating the effect of these dynamics on aquatic ecosystem functions.

## Supporting information

S1 Fig Total biomass anomalies by station. Total biomass anomalies of July-August mean total phytoplankton biomass.
(DOCX)

S2 Fig Mean July-August phytoplankton class biomass or biovolume time series by station. (DOCX)

S3 Fig Anomalies of mean July-August phytoplankton class biomass time series by station. These are z-scored biomass time series. Four major classes are shown–cyanobacteria, dinoflagellates (primarily auto- & mixotrophs), diatoms, and cryptophytes.
(DOCX)

S4 Fig Total biomass model trend and loadings. (DOCX)

S5 Fig Class time series observations and model predicted values. (DOCX)

S6 Fig Class model trends and loadings for model fit with 1990 and 2008 replaced with NA values. (DOCX)

S7 Fig Environmental times series. Temperature and salinity by station, NAO, and BSI time series.
(DOCX)

S1 Table Environmental time series overview and sources. (DOCX)

S2 Table Model selection for total biomass anomaly models. (DOCX)

S3 Table Class model trend and regional climate variable correlations. (DOCX)

S4 Table Class model time series residuals and local environmental variable correlations. (DOCX)
