# Supplementary material for: Limited evidence for common interannual trends in Baltic Sea summer phytoplankton biomass
Source: PLoS One. 2020 Apr 30;15(4):e0231690. doi: 10.1371/journal.pone.0231690 (PMC7192432; doi:10.1371/journal.pone.0231690)
Supplement: S1 Fig — Total biomass anomalies of July-August mean total phytoplankton biomass. (DOCX) [file pone.0231690.s001.docx]

Figure S1. Total biomass anomalies of July-August mean total phytoplankton biomass. Stations from top left to bottom right (by row) are ordered by geographic location (southwest to northeast)
