# Supplementary material for: Limited evidence for common interannual trends in Baltic Sea summer phytoplankton biomass
Source: PLoS One. 2020 Apr 30;15(4):e0231690. doi: 10.1371/journal.pone.0231690 (PMC7192432; doi:10.1371/journal.pone.0231690)

Figure S2. Mean July-August phytoplankton class biomass time series by station. Note y-axis ranges and units differ among stations. Units are those as provided by the source monitoring program listed in Table 1 in the manuscript.


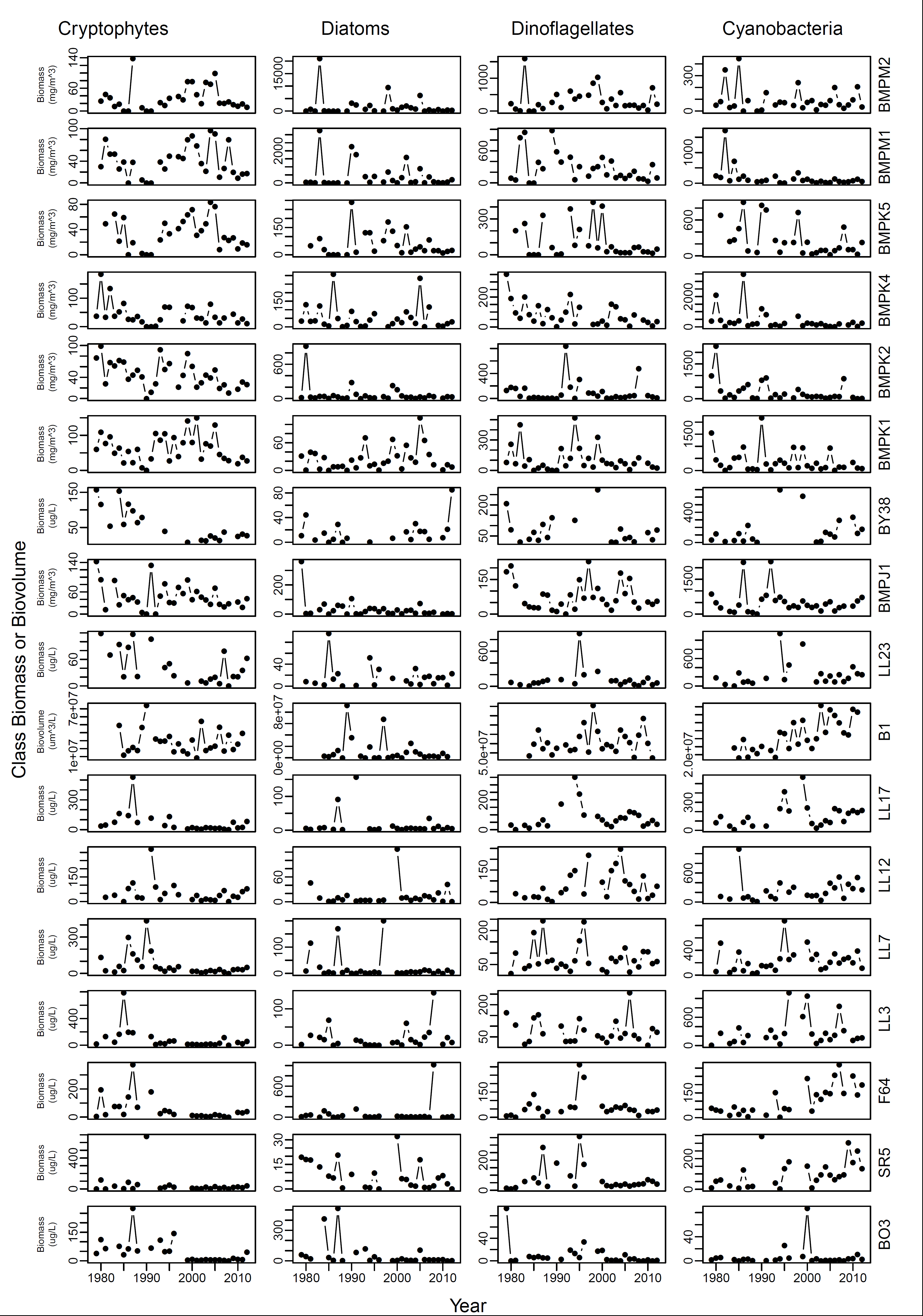

Supplement: S2 Fig — (DOCX) [file pone.0231690.s002.docx]
