# Supplementary material for: Limited evidence for common interannual trends in Baltic Sea summer phytoplankton biomass
Source: PLoS One. 2020 Apr 30;15(4):e0231690. doi: 10.1371/journal.pone.0231690 (PMC7192432; doi:10.1371/journal.pone.0231690)

Figure S3. Anomalies of mean July-August phytoplankton class biomass time series by station. These are z-scored biomass time series.


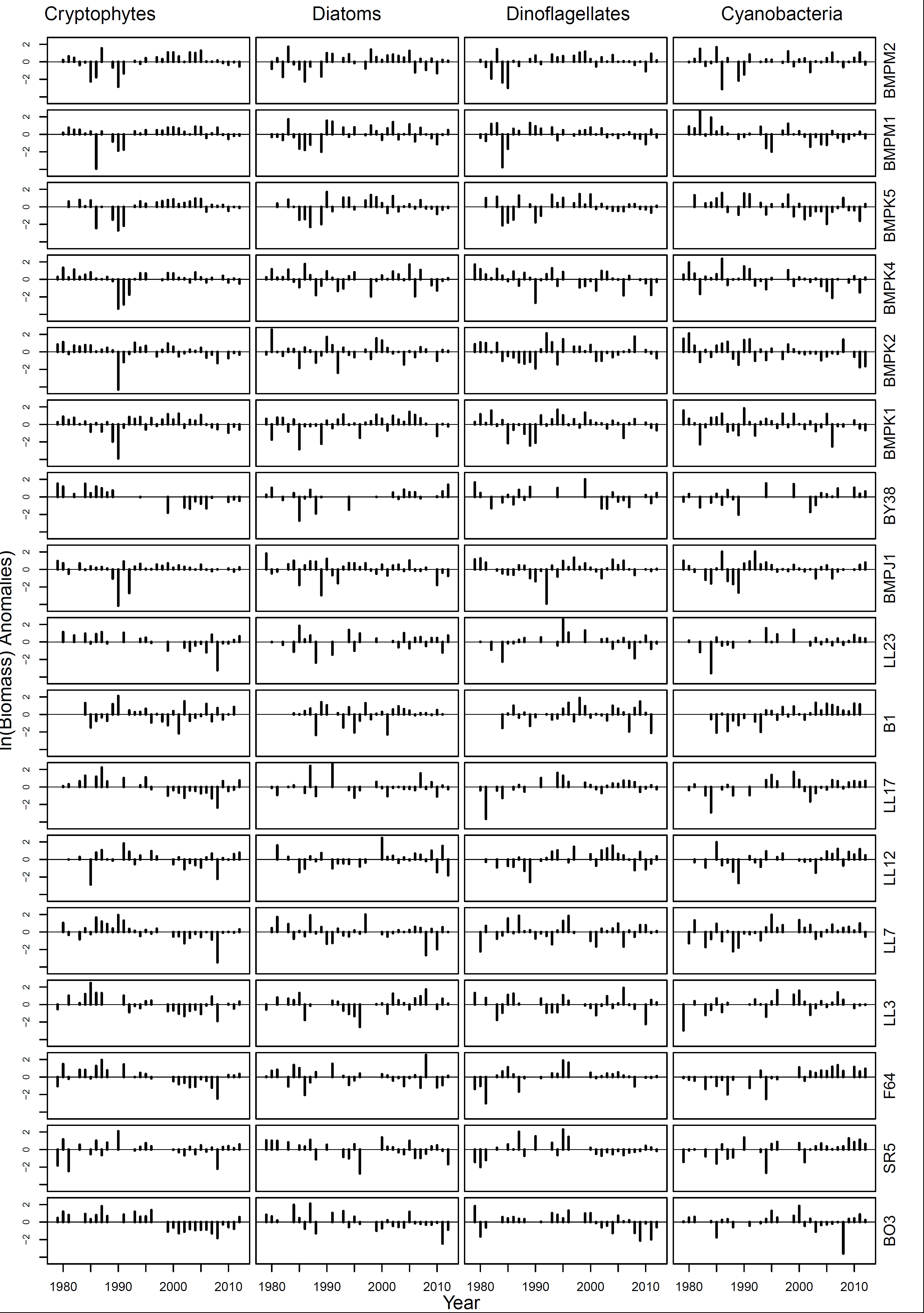

Supplement: S3 Fig — These are z-scored biomass time series. Four major classes are shown–cyanobacteria, dinoflagellates (primarily auto- & mixotrophs), diatoms, and cryptophytes. (DOCX) [file pone.0231690.s003.docx]
