# Supplementary material for: Limited evidence for common interannual trends in Baltic Sea summer phytoplankton biomass
Source: PLoS One. 2020 Apr 30;15(4):e0231690. doi: 10.1371/journal.pone.0231690 (PMC7192432; doi:10.1371/journal.pone.0231690)
Supplement: S5 Fig — (DOCX) [file pone.0231690.s005.docx]

Figure S5. Class time series observations and model predicted values (2 pages).

Figure S5. Continued
