# Supplementary figures and images for: Limited evidence for common interannual trends in Baltic Sea summer phytoplankton biomass
Source: PLoS One. 2020 Apr 30;15(4):e0231690. doi: 10.1371/journal.pone.0231690 (PMC7192432; doi:10.1371/journal.pone.0231690)

Figure S6. Class model trends and loadings for model fit with 1990 and 2008 replaced with NA values.


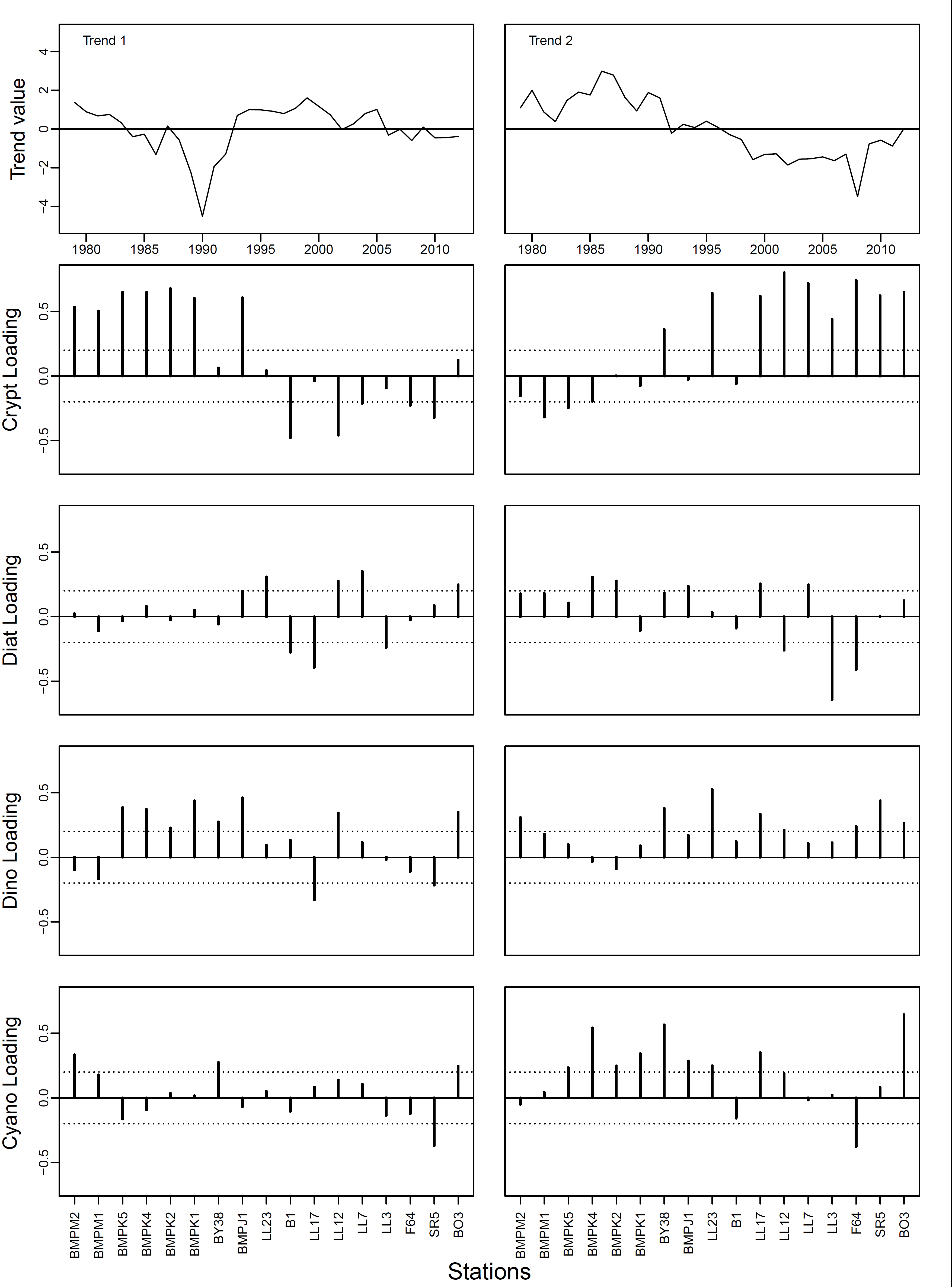

Supplement: S6 Fig — (DOCX) [file pone.0231690.s006.docx]
