# Supplementary material for: Limited evidence for common interannual trends in Baltic Sea summer phytoplankton biomass
Source: PLoS One. 2020 Apr 30;15(4):e0231690. doi: 10.1371/journal.pone.0231690 (PMC7192432; doi:10.1371/journal.pone.0231690)

Figure S7. July-August mean temperature (grey triangles) and salinity (black dots) time series by station. All stations are show data summarized from 0-10m except B1which is from 0-20m. The bottom right plot shows the winter NAO and July-August and winter BSI time series.


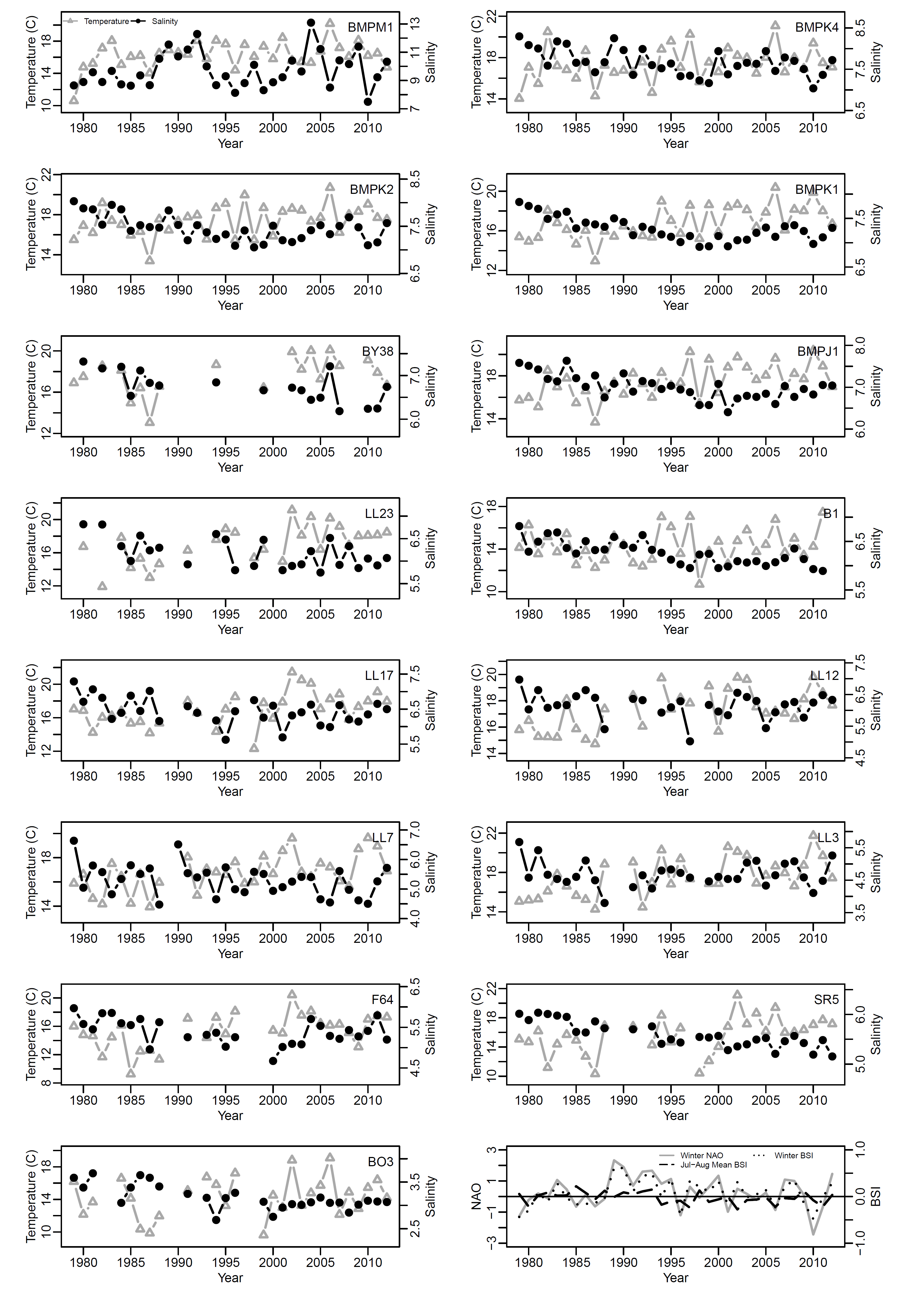

Supplement: S7 Fig — Temperature and salinity by station, NAO, and BSI time series. (DOCX) [file pone.0231690.s007.docx]
