# Supplementary material for: Limited evidence for common interannual trends in Baltic Sea summer phytoplankton biomass
Source: PLoS One. 2020 Apr 30;15(4):e0231690. doi: 10.1371/journal.pone.0231690 (PMC7192432; doi:10.1371/journal.pone.0231690)
Supplement: S1 Table — (DOCX) [file pone.0231690.s008.docx]

Table S1. Environmental Time Series Overview and Sources. Stations are ordered from southwest to northeast. BMP K5 and BMP M2 had no clear station associated with its sampling coordinates in the NEST system. Prior to 2008, data collected by SYKE/FMI were collected by Finnish Institute of Marine Research
