# Supplementary material for: Limited evidence for common interannual trends in Baltic Sea summer phytoplankton biomass
Source: PLoS One. 2020 Apr 30;15(4):e0231690. doi: 10.1371/journal.pone.0231690 (PMC7192432; doi:10.1371/journal.pone.0231690)
Supplement: S2 Table — (DOCX) [file pone.0231690.s009.docx]

Table S2. Model selection for total biomass anomaly models. Only models with dAICc < 20 are shown. R-structure is the variance-covariance matrix structure. Covariate coefficient structure indicates whether time series shared the same coefficient for covariate or each had a unique coefficient.
