# Supplementary material for: Limited evidence for common interannual trends in Baltic Sea summer phytoplankton biomass
Source: PLoS One. 2020 Apr 30;15(4):e0231690. doi: 10.1371/journal.pone.0231690 (PMC7192432; doi:10.1371/journal.pone.0231690)
Supplement: S3 Table — (DOCX) [file pone.0231690.s010.docx]

Table S3. Class model trend and regional climate variable correlations. Sample size (N) and adjusted sample size (Nstar) to give appropriate degrees of freedom given autocorrelation in the time series. The critical value is based on Nstar for a two-tailed test with α = 0.05
