# Supplementary material for: Limited evidence for common interannual trends in Baltic Sea summer phytoplankton biomass
Source: PLoS One. 2020 Apr 30;15(4):e0231690. doi: 10.1371/journal.pone.0231690 (PMC7192432; doi:10.1371/journal.pone.0231690)
Supplement: S4 Table — (DOCX) [file pone.0231690.s011.docx]

Table S4. Class model time series residuals and local environmental variable correlations
